# Comprehensive Literature Review of Obstetric Outcomes and Fetal Risk during Pregnancy with Pseudoxanthoma Elasticum

**DOI:** 10.3390/jcm10112532

**Published:** 2021-06-07

**Authors:** Raphael Lee, Mark Lebwohl

**Affiliations:** Department of Dermatology, Icahn School of Medicine at Mount Sinai, New York, NY 10029, USA; mark.lebwohl@mountsinai.org

**Keywords:** pseudoxanthoma elasticum, calcification, elastic fibers, pregnancy, placenta

## Abstract

Individuals with pseudoxanthoma elasticum (PXE) have often been advised against becoming pregnant due to a fear of the exacerbation of existing symptoms, likelihood of inheritance of the disease, and possible obstetric risks associated with the mother and child. PXE is a recessive multisystem disorder that leads to calcification of elastic tissues and fibers that can result in arterial rupture and gastrointestinal (GI) bleeding, possibly endangering the fetus and mother. PXE often manifests in skin lesions as well and the risk of exacerbation is a principal concern. To address these complications and to provide transparent understanding to healthcare providers and mothers of the associated risk factors with pregnancy and PXE, we conducted a comprehensive review of the current literature and found that there is no inherent risk for obstetric complications for PXE pregnancies and patients need not be advised against becoming pregnant as previously suggested. PXE-related pregnancies are unremarkable to the mother’s wellbeing and fetal complications are few, if any at all.

## 1. Introduction

Pseudoxanthoma elasticum (PXE) is a progressive multisystem autosomal disorder characterized by calcification of elastic fibers that impact the integrity and strength of connective tissues of the skin, eye, and vascular system of the body. Arteries within the urogenital tract, which are lined by an internal elastic lamina, can become calcified, leading to narrowing and subsequent breakage and internal bleeding. Further research has localized the underlying genetic cause to the ABCC6 gene on chromosome 16p13.1 [1]. Deleterious inactivating mutations of the ABCC6 gene lead to chronically decreased levels of plasma inorganic pyrophosphate concentrations, resulting in increased levels of vascular calcification in addition to higher risk of cardiovascular issues and PXE [2].

It has been illustrated that inorganic pyrophosphate, a vascular mineralization inhibitor, acts as a substrate to non-specific alkaline phosphatase (TNAP) which cleaves inorganic pyrophosphate into inorganic phosphate, inactivating its catalytic inhibitory activity. TNAP has been shown to be heavily expressed in the placenta, in addition to being at elevated levels during gestation, consequently leading to decreased levels of plasma inorganic pyrophosphate concentrations [2]. Additionally, the uterus has been shown to possess elastic fibers, functionally serving to relieve excess pressure on the growing fetus [3]. While not yet fully understood, it is posited that the regular increase of TNAP activity during pregnancy is linked to normal fetal development and growth. For patients with PXE, the levels of plasma inorganic pyrophosphate are characteristically low ab ovo, putting these patients at possibly higher risk of developing vascular issues and bleeds with the advent of gestation since the increase in TNAP activity results in even lower than baseline inorganic pyrophosphate concentrations. Historically, the main concern for PXE and pregnancy was the risk of GI bleeding and possible fetal complications.

For these reasons, we performed a review of the most recent and current literature on PXE in relation to obstetric complications and outcomes to identify the presence of possible risks, disease prognosis and manifestation, and fetal impact and outcomes for PXE-afflicted individuals of childbearing potential.

## 2. Materials and Methods

An online literature search was performed at the Icahn School of Medicine at Mount Sinai Health System, providing access to the following online databases: PubMed, Google Scholar, NCBI, NORD, and Wiley Online Library. The search was conducted between January 2021 to February 2021, using keywords “placental calcification”, “pseudoxanthoma elasticum and pregnancy”, and “PXE and pregnancy”.

Due to the rare nature and low incidence rate of the disease (~1 per 100,000 people), in addition to the consequent lack of extensive literature in relation to pregnancy, there was no criteria set for the publication date and all relevant articles were consulted. The publication dates for the articles range from 1984 to the most recent at 2020. Only articles and reviews in English were consulted.

## 3. Results

Through our search, we reviewed 21 articles on PXE and pregnancy. Ten were case reports dating from 1984 to 2014. Four were series (multiple cases). Six were review or basic research articles used for reference that mention pregnancy but did not report new cases. One was a comprehensive literature review conducted in 2016. A summary of the clinically relevant results are included in Table S1.

It should be noted that the rarity of PXE and the lack of extensive literature on PXE in relation to pregnancy leads to the majority of the articles being individually reported case reports and not clinical trials or longitudinal analytical studies. This limitation naturally leads to less comprehensive and definitive analyses, as any large claim will be difficult to substantiate due to the data being drawn from isolated incidents and reports.

Of the reported outcomes, the most frequently reported maternal complications were worsening manifestation of skin such as the formation of abdominal striae (16%), hypertension (9.5%), and skin lesion aggravation (0.4%). Of those reported complications, only the aggravation of skin lesions, particularly around the neck and abdomen, and cosmetic deterioration of the abdominal skin could be directly related to PXE in nature. That said, the development of abdominal striae is a common feature across many pregnancies that appears positively correlated with maternal weight gain and multiparity and cannot be directly classified as a PXE symptom aggravation [4]. Less common complications due to PXE such as angioid streaks and a single report of acute GI bleeding were also reported. However, the patient with the GI bleeding reported an extensive history of acute GI bleeds independent of pregnancy and thus could not be necessarily be attributed as a consequence of PXE and gestation [5]. The acute GI bleed occurred during gestation but the impact on obstetric outcome and delivery complications were not reported in the case study. Additionally, there were no reports of PXE aggravation or increased calcification of the uterine tissue, which has been shown to possess elastic fibers [3].

In terms of fetal complications, calcification of the placenta was the most common PXE manifestation occurring in 60% of reported cases. Nonetheless, there was a negligible impact on the fetus as the majority of all cases led to healthy births via caesarean section or vaginal delivery. Of the reported cases, one case mentioned decelerated fetal growth during development at around week 26, indicating the need for an emergency caesarean section, but was uneventful as the healthy infant caught up to normal growth by six weeks post-delivery [6]. Another case report highlights newborn twins who suffered from intraventricular hemorrhage due to maternal bleeding at week 26, although it stands in isolation as the only case with a poor fetal outcome.

## 4. Discussion

Patients with PXE often have great hesitation towards becoming pregnant for two main reasons: the first being the risk of inheritance of PXE to the child given the genetic nature of the disease, and the second, the possibility of exacerbating PXE symptoms that lead to poor outcomes for both the mother and infant.

PXE is an autosomal recessive trait and is very uncommon within the gene pool with a frequency of 1 per 56,000; thus, it would be highly unlikely that the next generation would have the disease unless there is consanguinity between the parents [7]. The disease follows a predictable autosomal recessive inheritance pattern [1]. Additionally, the gene has been identified and genetic testing and counseling is available to identify any possible risk of inheritance. With such technologies, patients with PXE are able to transparently understand their options and assess the associated risk of inheritance. Due to the extreme rarity of the disease and our meta-analysis of the most recent literature, the likelihood of inheritance of PXE is minimal as none of the infants reported symptoms of PXE manifestation. Still, PXE is a late-onset disease that goes unnoticed and usually does not manifest until the second decade of life [8]. PXE should be monitored for as the case reports analyzed did not conduct longitudinal follow-ups with the infants, though some do briefly mention that the infants grew up to become healthy adults [4].

From our review of the current literature, there seems to be no inherent risk of obstetric complications for women with PXE as opposed to their non-PXE counterparts. PXE-specific complications such as GI bleeding and angioid streaks are less common than previously thought, occurring in 3 out of 10 of the analyzed case reports, while more frequently reported complications such as striae and hypertension occurred more often but are easily manageable. Due to the complete absence of bleeding problems across 54 PXE-related pregnancies in their analysis, Viljoen stated that previously reported hemorrhagic complications—such as the historic concern of GI bleeding—were most likely over-reported in comparison to normal pregnancies [4]. From our own analysis of the current literature, we agree with this statement. This conclusion appears confidently maintained across all of the studies that included multiple reported cases as normal pregnancies heavily outnumber pregnancies that report both PXE specific and non-PXE complications.

The worsening of pre-existing angioid streaks and the advent of new ones was argued to serve as indications for pursuing medical interventions during delivery, but this symptom manifestation does not seem to worsen fetal or maternal outcomes. The development of angioid streaks do not affect visual acuity, nor do they necessitate caesarean section alone, although the development of active choroidal neovascularization must be monitored for as vaginal delivery could be contraindicated for such cases [8].

From our own analysis, maternal age appears to have no impact on the severity and clinical manifestation of PXE complications during gestation and delivery, although obstetric and fetal complications naturally increase in risk with advanced maternal age within the general population. To assess this, we looked into maternal age and reported obstetric and fetal complication frequency to see if any association or correlation could be derived. However, a recent 2020 study looking into cardiovascular disease risk and inorganic pyrophosphate levels found that multiparous PXE patients reported significantly higher normalized lower limb artery calcification scores as compared to uni/nulliparous PXE patients in relation to advancing maternal age. Given that patients with PXE have decreased inorganic pyrophosphate levels to begin with, older multiparous women have been shown to aggregate greater levels of alkaline phosphatase activity with each subsequent child. The positive correlation leads to higher levels of vascular calcification, a known factor for cardiovascular disease risk [2]. Diligent monitoring of cardiovascular disease risk should be considered for providers working with older multiparous women with PXE. While there are some side effects to PXE that can occur, most pregnancies are unremarkable from conception through birth other than extensive placental calcification, which was present in almost every single case.

Due to the lack of poor outcomes of newborn infants, the calcification of the placenta has minimal physiological consequences as the mineralization does not seem to occlude any transport of nutrients and resources between the maternal–fetal boundary. Additionally, fetal impact seems to be minimal as the majority of pregnancies are carried fully to term without any complications during delivery and without any developmental issues for the infant. Decreased fetal development around week 26 of gestation was noted in two cases, but both instances were unremarkable in the end as one was quickly resolved upon maternal rest and the other caught up to expected healthy infant weight six weeks post-birth. As such, intrauterine growth retardation seems unlikely given that only 2 out of 923 pregnancies analyzed in this review resulted in decelerated fetal development. Still, previous studies report that rare instances of severe septal calcification and intense echogenicity could be indications of fetal growth retardation and should be monitored for if noted during gestation. Infants born to PXE mothers are at no greater risk of complications throughout pregnancy and delivery. Granted, no studies analyzed conducted longitudinal follow-ups with infants born from PXE-pregnancies to confirm the absence of PXE-originated health issues and complications later in life.

Many of the papers reviewed were isolated case reports that indicate some possible complications that have occurred; however, being case reports, the frequency of such complications cannot be treated as definitive across all pregnancies. Even so, the majority of those case reports reviewed led to unremarkable births and healthy infants with no issue for the mother in the end. Perhaps most illustrative of our findings is Bercovitch’s 2004 study in which 795 (*n* = 306) PXE-related pregnancies were investigated. The Bercovitch study provided the largest sample size for PXE and pregnancy research, allowing for more complete and applicable analysis and conclusions to be drawn that applies to the greater population. Through his research and analysis, Bercovitch found that there is no more risk of complications for PXE pregnancies as there are for non-PXE pregnancies, and that PXE’s historical concern of retinal complications and gastric bleeding for expectant mothers occurred in less than 1% of all pregnancies [9]. The most present complication reported was worsened skin manifestations (12%) followed by hypertension (10%), both of which do not critically endanger mother or child and can be easily addressed and monitored. Additionally, Bercovitch concludes that there is no increased risk of first-trimester miscarriage in PXE patients and that the incidence of uterine bleeding is well within the expected frequency of the normal population [9]. It is worth mentioning that three-quarters of all investigated PXE pregnancies within Bercovitch’s study reported no complications whatsoever.

## 5. Conclusions

From our comprehensive review of the current literature on PXE and pregnancy, we conclude that there is no greater risk for complications for pregnant PXE-afflicted women compared to women without PXE. Hesitations due to genetic inheritance can be addressed via genetic testing and counseling, while worsened clinical manifestations of PXE are uncommon. Maternal age should be considered in cases of multiparity, though the impact seems minimal. Although it is not necessary for individuals with PXE considering pregnancy to worry about possible obstetric complications and the aggravation of PXE-related symptoms, it is prudent for healthcare providers to recognize and monitor the management of such individuals with an emphasis on cardiac, vascular, and retinal care. A multidisciplinary team would best benefit the patient’s reproductive journey.

## Supplementary Materials

The following are available online at https://www.mdpi.com/article/10.3390/jcm10112532/s1: Table S1: Obstetric and Fetal Outcomes in Relation to PXE.
