# Supplementary material for: Comprehensive Literature Review of Obstetric Outcomes and Fetal Risk during Pregnancy with Pseudoxanthoma Elasticum
Source: J Clin Med. 2021 Jun 7;10(11):2532. doi: 10.3390/jcm10112532 (PMC8201327; doi:10.3390/jcm10112532)
Supplement: Supplementary file 1 [file jcm-10-02532-s001.zip › jcm-1170782-supplementary.pdf]

Table S1. Obstetric and Fetal Outcomes in Relation to Pseudoxanthoma Elasticum.

Author	Year	Sample Size	Maternal Age	Maternal/PXE Complications	Fetal Complications	Labor/Delivery Complications	Impact on Child
Elejalde B, et al. [1]	1984	1	30	- increased number of septa and abnormal elastic tissue	- deceleration of fetal growth post week 26<br>- heavy placental calcification	- labor induced due to severe intrauterine growth retardation	- none; infant delivered at 36 weeks with normal growth by 6 wks
Viljoen D, et al. [2]	1987	20 (54 pregnancies)	15–64	- abdominal striae in all patients (varying severity with multiparas and weight)<br>- hypertension in 7 pregnancies	- 12 miscarriages (between 6–14 weeks, unspecified)<br>- 2 early terminations (due to psychiatric reasons)	- none	- none; 40 live births (3 preterm)<br>- 1 infant died at 4 days of age due to complex congenital cardiac anomaly
Mansat-Krzyzanowska E, et al. [3]	1993	1		- skin lesion aggravation 2 years post partum	- transient inflection of fetal growth curve with return to normal after maternal rest<br>- placental calcification	- none<br>- caesarean section at week 35	- none; healthy infant delivered
Valenzano M, et al. [4]	2000	1	29	- skin lesion aggravation	- none	- none<br>- caesarean section at week 35	- none; healthy infant delivered
Gheduzzi D, et al. [5]	2001	29 (15 PXE, 14 control)	25–37	- 1 case of mild preeclampsia<br>- 1 case of hypertension<br>- 2 cases of first trimester spotting	- placental calcification	- none	- none; all newborns healthy
Zachariah M, et al. [6]	2003	1	25	- worsened skin manifestation<br>- skin lesion aggravation<br>- prominent abdominal striae<br>- hypertension at week 38	- none	- labor induced due to pre-eclampsia<br>- caesarean section required because of secondary arrest at 5 cm dilatation	- none, healthy infant delivered
Bercovitch L, et al. [7]	2004	306 (795 pregnancies)	15–41	- hypertension (10%)<br>- gastric bleeding (<1%)<br>- retinal complication (<1%)<br>- worsened skin manifestation (12%)	- 8 stillbirths (1 due to maternal PXE GI bleeding)	- caesarean section (13% of deliveries)	- none; 660 live births
Xiromeritis P, et al. [8]	2006	1(2 pregnancies)	28	- worsened skin manifestation on neck	- placental calcification	- vacuum extraction for first child	- none; both infants delivered and healthy
Fahri D, et al. [9]	2006	1	25	- worsening of cutaneous lesions<br>- development of severe angioid streaks	- none	- caesarean section due to angioid streaks	- not reported
Goral V, et al. [10]	2007	1	28	- acute gastrointestinal hemorrhage (patient has extensive history of GI bleeding)	- none reported	- none reported	- not reported
Ching Tan W, et al. [11]	2008	1	37	- none	- markedly echogenic placenta due to extensive calcification	- none<br>- elective caesarean section	- none; healthy infant delivered at 38 weeks with normal growth at 6 months
Tanioka M, et al. [12]	2014	1	26	- none	- severe placental calcification at week 30	- none	- none; healthy infant delivered at 39 weeks
Drue HC, et al. [13]	2014	1	36	- maternal hemorrhage at 27 weeks	- hyperechogenic placental calcification	- caesarean section due to maternal bleeding at 27 weeks	- both twins suffered from intraventricular hemorrhage due to maternal bleeding

Veiga-Lopez A, et al. [14]	2020	48	40+	- multiparous women (>2 births) had significantly higher normalized lower limb artery calcification score compared to uni/nulliparous	- not reported	- not reported	- not reported

References

1. Elejalde, B.R.; De Elejalde, M.M.; Samter, T.; Burgess, J.; Lombardi, J.; Gilbert, E.F.; Opitz, J.M.; Reynolds, J.F. Manifestations of pseudoxanthoma elasticum during pregnancy: A case report and review of the literature. *Am. J. Med. Genet.* **1984**, *18*, 755–762, doi:10.1002/ajmg.1320180422.
2. Viljoen, D.L.; Beatty, S.; Beighton, P. The obstetric and gynaecological implications of pseudoxanthoma elasticum. *BJOG Int. J. Obstet. Gynaecol.* **1987**, *94*, 884–888, doi:10.1111/j.1471-0528.1987.tb03760.x.
3. Mansat-Krzyzanowska, E.; Sagot, P.; Le Neel, N.; Stalder, J.F. Pseudoxanthome élastique et grossesse [Pseudoxanthoma elasti-cum and pregnancy]. *Ann. Dermatol. Venereol.* **1993**, *120*, 391–394.
4. Valenzano, M.; Corticelli, A.; Podestà, M.; Nicoletti, L.; Saffioti, S.; Derchi, L. Pseudoxanthoma elasticum and pregnancy: A case report. *Clin. Exp. Obstet. Gynecol.* **2000**, *27*, 11214955.
5. Gheduzzi, D.; Taparelli, F.; Quaglino, D.; Di Rico, C.; Bercovitch, L.; Terry, S.; Singer, D.; Pasquali-Ronchetti, I. The Placenta in Pseudoxanthoma Elasticum: Clinical, Structural and Immunochemical Study. *Placenta* **2001**, *22*, 580–590, doi:10.1053/plac.2001.0687.
6. Zachariah, M.; Thomas, S.B.; Stokes, I. Pseudoaxanthoma elasticum and pregnancy. *J. Obstet. Gynaecol.* **2003**, *23*, 433–434, doi:10.1080/0144361031000122606.
7. Bercovitch, L.; Leroux, T.; Terry, S.; Weinstock, M. Pregnancy and obstetrical outcomes in pseudoxanthoma elasticum. *Br. J. Dermatol.* **2004**, *151*, 1011–1018, doi:10.1111/j.1365-2133.2004.06183.x.
8. Xiromeritis, P.; Valembois, B. Pseudoxanthoma elasticum and pregnancy. *Arch. Gynecol. Obstet.* **2005**, *273*, 253–254, doi:10.1007/s00404-005-0075-1.
9. Farhi, D.; Descamps, V.; Picard, C.; Mahieu-Caputo, D.; Doan, S.; Crickx, B. Is pseudoxanthoma elasticum with severe angioid streaks an indication for Caesarean section? *J. Eur. Acad. Dermatol. Venereol.* **2006**, *20*, 1361–1362, doi:10.1111/j.1468-3083.2006.01717.x.
10 Goral, V.; Demir, D.; Tuzun, Y.; Keklikci, U.; Buyukbayram, H.; Bayan, K.; Uyar, A. Pseudoxantoma elasticum, as a repetitive upper gastrointestinal hemorrhage cause in a pregnant woman. *World J. Gastroenterol.* **2007**, *13*, 3897–3899, doi:10.3748/wjg.v13.i28.3897.
11 Tan, W.C.; Rodeck, C.H. Placental calcification in pseudoxanthoma elasticum. *Ann. Acad. Med. Singap.* **2008**, *37*, 598–600.
12 Tanioka, M.; Utani, A.; Tamura, H.; Yoshimura, N.; Kashiwagi, N.; Kondo, E.; Konishi, I.; Miyachi, Y. Calcification of the placenta in a woman with pseudoxanthoma elasticum with a mutation of theABCC6gene. *J. Dermatol.* **2014**, *41*, 189–191, doi:10.1111/1346-8138.12360.
13 Drue, H.C.; Mogensen, H.; Olesen, A.W. Pregnancy jeopardized by pseudoxanthoma elasticum. *Ugeskr. Laeger.* 2014 Oct 6;176(41):V04140206. Danish. PMID: 25331665.
14 Veiga-Lopez, A.; Sethuraman, V.; Navasiolava, N.; Makela, B.; Olomu, I.; Long, R.; Van De Wetering, K.; Martin, L.; Aranyi, T.; Szeri, F. Plasma Inorganic Pyrophosphate Deficiency Links Multiparity to Cardiovascular Disease Risk. *Front. Cell Dev. Biol.* **2020**, *8*, 573727, doi:10.3389/fcell.2020.573727.
